# Spindle Dynamics Model Explains Chromosome Loss Rates in Yeast Polyploid Cells

**DOI:** 10.3389/fgene.2018.00296

**Published:** 2018-08-06

**Authors:** Ivan Jelenić, Anna Selmecki, Liedewij Laan, Nenad Pavin

**Affiliations:** ^1^Department of Physics, Faculty of Science, University of Zagreb, Zagreb, Croatia; ^2^Department of Medical Microbiology and Immunology, Creighton University Medical School, Omaha, NE, United States; ^3^Department of Bionanoscience, Faculty of Applied Sciences, Kavli Institute of NanoScience, Delft University of Technology, Delft, Netherlands

**Keywords:** polyploidy, spindle assembly, chromosome loss, chromosome segregation, cell cycle regulation, theoretical modeling, genome instability

## Abstract

Faithful chromosome segregation, driven by the mitotic spindle, is essential for organismal survival. Neopolyploid cells from diverse species exhibit a significant increase in mitotic errors relative to their diploid progenitors, resulting in chromosome nondisjunction. In the model system *Saccharomyces cerevisiae*, the rate of chromosome loss in haploid and diploid cells is measured to be one thousand times lower than the rate of loss in isogenic tetraploid cells. Currently it is unknown what constrains the number of chromosomes that can be segregated with high fidelity in an organism. Here we developed a simple mathematical model to study how different rates of chromosome loss in cells with different ploidy can arise from changes in (1) spindle dynamics and (2) a maximum duration of mitotic arrest, after which cells enter anaphase. We apply this model to *S. cerevisiae* to show that this model can explain the observed rates of chromosome loss in *S. cerevisiae* cells of different ploidy. Our model describes how small increases in spindle assembly time can result in dramatic differences in the rate of chromosomes loss between cells of increasing ploidy and predicts the maximum duration of mitotic arrest.

## Introduction

Chromosome segregation is an important, highly conserved cellular function. A complex network of interacting components segregates chromosomes with high precision. However, rare errors in chromosome segregation are observed, and the error rate generally increases when the number of sets of chromosomes (ploidy, *n*) increases within the cell (Comai, 2005). Increased rates of chromosome loss are observed in autopolyploid cells, within yeasts, plants, and human cells (Mayer and Aguilera, 1990; Song et al., 1995; Ganem et al., 2009). For example, autopolyploidization of *Phlox drummondii* results in an immediate loss of approximately 17% of genomic DNA in the first generation and up to 25% after three generations (Raina et al., 1994). Autopolyploidization can also cause tumorigenesis, and these tumors are marked by significant chromosome gain/loss events (Fujiwara et al., 2005; Zack et al., 2013). Therefore, the general observation is that many newly formed polyploid cells have increased chromosome segregation errors relative to isogenic diploid cells, and the cause of these errors is not known.

The normal sexual life cycle of the budding yeast *Saccharomyces cerevisiae* includes haploid (*n* = 1, 16 chromosomes) and diploid cells (*n* = 2, 32 chromosomes). In addition, tetraploid cells (*n* = 4, 64 chromosomes) are rarely found in nature, but can be generated in the lab by mating two diploid cells. In this organism, the effect of ploidy on the rate of chromosome loss is very pronounced: haploid and diploid cells have rates of chromosome loss around 10^−6^ chromosomes per cell per cell division, whereas tetraploid cells have a rate around 10^−3^ (Mayer and Aguilera, 1990; Storchová et al., 2006). The rate of chromosome loss was measured with isogenic haploid, diploid, and tetraploid strains that each contained a single genetically marked chromosome. In these assays the cells that have lost the chromosome markers are quantified, and the rate of loss is determined by fluctuation analysis (Lea and Coulson, 1949). Moreover, polyploid laboratory yeast strains tend to lose chromosomes and reduce to a diploid level in experimental evolution studies (Gerstein et al., 2006; Selmecki et al., 2015). Thus, the genomic stability of a cell line is to a large extent related to cellular ploidy, but how ploidy alters chromosome segregation is not known (Otto and Whitton, 2000).

Chromosome segregation is driven by the mitotic spindle, a self-organized micro-machine composed of microtubules and associated proteins (Pavin and Tolić, 2016; Prosser and Pelletier, 2017). In budding yeast, during spindle assembly, spindle poles nucleate microtubules, which grow in a direction parallel with the central spindle or in arbitrary directions within the nucleus (Winey et al., 1995; O'Toole et al., 1997). A microtubule that comes into the proximity of a kinetochore (KC), a protein complex at the sister chromatids, can attach to the KC and thus establish a link between chromatids and spindle poles, as shown *in vitro* (Mitchison and Kirschner, 1985; Akiyoshi et al., 2010; Gonen et al., 2012; Volkov et al., 2013), *in vivo* (Tanaka et al., 2005), and theoretically (Hill, 1985). Theoretical models have quantitatively shown that this process can contribute to spindle assembly in yeasts and in mammalian cells (Wollman et al., 2005; Paul et al., 2009; Kalinina et al., 2013; Vasileva et al., 2017). Prior to chromosome separation, all connections between chromatids and the spindle pole must be established, and erroneous KC-microtubule attachments must be corrected, for which several theoretical models have been proposed (Zaytsev and Grishchuk, 2015; Tubman et al., 2017). These connections are monitored by the spindle assembly checkpoint (Li and Murray, 1991). Once KCs are properly attached and chromosomes congress to the metaphase plate (Gardner et al., 2008), the spindle assembly checkpoint is silenced and microtubules separate the sister chromatids (Musacchio and Salmon, 2007).

Cells that cannot satisfy the spindle assembly checkpoint are arrested in mitosis. However, cells can break out of the arrest after several hours, an event that is often referred to as “mitotic slippage” (Minshull et al., 1996; Rudner and Murray, 1996; Rieder and Maiato, 2004), and this mitotic exit is molecularly regulated (Novák et al., 1999; Rudner et al., 2000). Even though the molecular mechanisms that regulate cell cycle and spindle assembly are emerging, it is an open question as to how changes in ploidy can have such a dramatic effect on the rates of chromosome loss.

In this paper, we introduce a theoretical model for chromosome loss in cells with different ploidy. We test the hypothesis that polyploidy limits faithful chromosome segregation by the combination of dynamics of spindle assembly and a maximum time of mitotic arrest. Our model predicts that for increasing ploidy, spindle assembly time scales linearly with the number of chromosomes, which results in exponential changes in the rate of chromosome loss. Our model quantitatively reproduces the increase in chromosome loss observed in tetraploid *S. cerevisiae* cells relative to haploid and diploid cells.

## Materials and methods

### Model for chromosome loss

**Figure 1:**
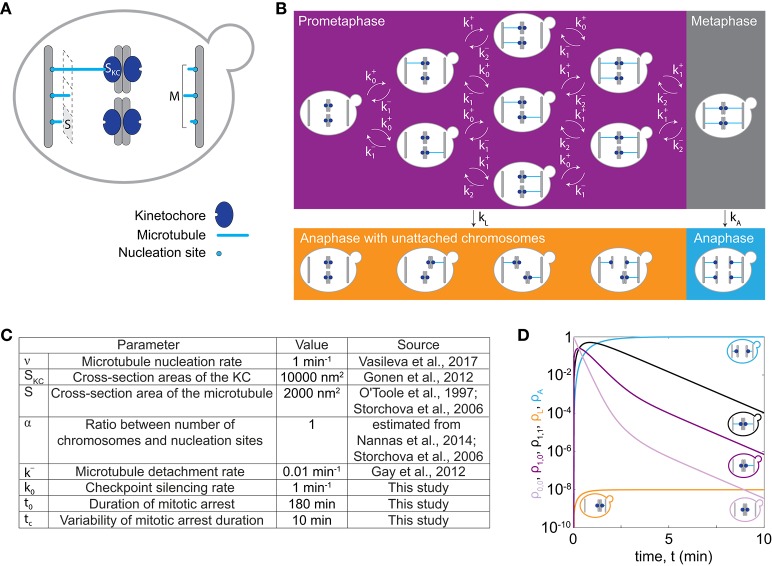
Model for chromosome loss. **(A)** Spindle geometry in an individual cell. A microtubule (light blue) occupies a cross-section area *S*. Microtubules nucleate from *M* nucleation sites at the spindle pole body (gray bar) and extend toward KCs (dark blue) of a cross-section area *S*_*KC*_. **(B)** Spindle dynamics in mitosis. The different boxes indicate cells in prometaphase (purple box), metaphase (gray box) and anaphase (orange and blue box). Arrows denote the rate of transition between different populations. Within a cell, microtubules (blue lines) extend from the spindle pole bodies (gray bars) toward the KCs (dark blue circles). **(C)** Parameters used to solve the model. Five parameter values were taken from previous studies (O'Toole et al., 1997; Storchová et al., 2006; Gay et al., 2012; Gonen et al., 2012; Nannas et al., 2014; Vasileva et al., 2017), as indicated. **(D)** Solution of the model for cells with 1 chromosome (*C* = 1). Fraction of cells in prometaphase with no KCs attached (light purple, ρ_0,0_), with 1 KC attached (dark purple, ρ_1,0_ or ρ_0,1_), in metaphase (black, ρ_1,1_), in anaphase with at least one KC unattached (orange, ρ_*L*_) and in anaphase (blue, ρ_*A*_), are shown. Each line is accompanied by a cell cartoon depicting the corresponding phase of the cell cycle. At *t* = 0, ρ_0,0_ = 1 and all other populations are 0.

In our model we describe the dynamics of spindle assembly including KC attachment and detachment (Figure 1A), silencing of the spindle assembly checkpoint and the maximum duration of mitotic arrest after which cells enter anaphase regardless of whether all KCs are attached, allowing for chromosome loss in our model. To make a prediction for chromosome loss, we describe populations of cells in prometaphase, metaphase, and anaphase with either all KCs attached to the spindle, or with at least one unattached KC, and we calculate the fraction of cells in each population (Figure 1B). Transitions between these populations arise from spindle dynamics (Figure 1A).

#### Dynamics of spindle assembly

To describe dynamics of spindle assembly, we calculate the rate of KC capture, $k_{i}^{+}$, by taking into account known microtubule dynamics and geometry of yeast spindles (Figure 1A). Here, index *i* denotes the number of left sister KCs attached to the spindle; analogous calculations are applied to right sister KCs. Microtubules nucleate from the spindle pole body at rate ν_*i*_ and extend toward the spindle equator. They can attach to an unattached KC with probability *p*. The rate of KC attachment is the probability of attachment of one of the unattached KCs multiplied with the microtubule nucleation rate, which for *C* chromosomes and *C*−*i* unattached KCs reads

(1) $$\begin{array}{r} k_{i}^{+}=\left[1-{\left(1-p\right)}^{C-i}\right]\nu_{i},\;i=0,\ldots ,C-1. \end{array}$$

For other values of the index *i* the rate of KC attachment is zero to exclude unrealistic cases, with a negative number of chromosomes or with more than *C* chromosomes. In the case of euploid cells, the number of chromosomes is related to the ploidy as *C* = 16 · *n*. We calculate the nucleation rate at the spindle pole body as *v*_*i*_ = *v* · *(M*−*i)*, where we assume that a spindle pole body has a constant number of *M* nucleation sites with *M*−*i* unoccupied nucleation sites. To determine *M* for different numbers of chromosomes, we introduce a linear relationship between the number of chromosomes and nucleation sites, *M* = α · *C* + 4, which is based on experimental findings (Storchová et al., 2006; Nannas et al., 2014). The parameter α is typically around 1. We also assume the nucleation rate for one nucleation site, ν , to be constant as in previous studies (Kitamura et al., 2010; Vasileva et al., 2017). In our model, attachment occurs when a microtubule contacts the KC (Tanaka et al., 2005). The probability of attachment is calculated based on spindle geometry as the ratio of the cross-section areas of the KC, *S*_*KC*_, and the total area of the spindle, *p* = *S*_KC_/(*S* · *M* + *S*_KC_). Here *S* denotes the cross-section area occupied by one microtubule. Values for these parameters are estimated from electron microscopy studies (O'Toole et al., 1997; Storchová et al., 2006; Gonen et al., 2012). We assume that microtubules detach from one KC at constant detachment rate, *k*^−^, because our model does not include forces at the KC (Akiyoshi et al., 2010).

#### Silencing the spindle assembly checkpoint and chromosome loss

Cells proceed from metaphase to anaphase by silencing the spindle assembly checkpoint at a constant rate, *k*_0_. They can also proceed from prometaphase to anaphase when they spend a prolonged time in mitotic arrest (Minshull et al., 1996; Rudner and Murray, 1996; Rieder and Maiato, 2004), which in our model results in chromosome loss. We distinguish these two cases by introducing a rate of anaphase entry given by

(2) $$\begin{array}{r} \left\{\begin{array}{c} k_{L}\\ k_{A} \end{array}\right\}=k_{0}\left\{\begin{array}{c} f\left(t\right)\\ 1+f\left(t\right) \end{array}\right\}, \end{array}$$

where in the top and bottom row we calculate rates at which cells leave prometaphase and metaphase, respectively. We describe bypassing the checkpoint in mitotic arrest with a function of time *f*(*t*), irrespective whether cells are in prometaphase or metaphase. Because this function is not known, we choose a simple mathematical form *f*(*t*) = *exp*[(*t* − *t*_0_)/*t*_*c*_], which accounts for the rate of anaphase entry increase in time. Here, parameters *t*_0_ and *t*_*c*_ denote the duration of mitotic arrest and the characteristic timescale, respectively.

#### Fraction of cells in prometaphase, metaphase, and anaphase with and without lost chromosomes

In our model, we denote the fractions of cells in prometaphase and metaphase by ρ_*i,j*_. The fraction of cells in anaphase with at least one KC unattached to the spindle, ρ_*L*_, represents the fraction with lost chromosomes. The fraction of cells in anaphase with all KCs attached is denoted ρ_*A*_. The indices *i* and *j* denote the number of left and right sister KCs attached to the spindle, respectively, in cells with *C* chromosomes (*i* = 0, …, *C* and *j* = 0, …, *C*). The combination of indices *i* = *j* = *C* describes cells with all KCs attached, which corresponds to metaphase cells. All the other combinations of indices describe cells with at least one unattached KC, which correspond to prometaphase cells. As time, *t*, progresses (i) KCs attach to or detach from the spindle, or (ii) cells enter anaphase changing the factions of cells in the populations (Figure 1B). In our model, attachments of different KCs as well as their detachments are independent. We describe these processes by a system of rate equations:

(3) $$\begin{array}{r} \frac{d\rho_{i,j}}{dt}=\;k_{i-1}^{+}\rho_{i-1,j}\;+\;k_{j-1}^{+}\rho_{i,j-1}\;+\;(i+1)k^{-}\rho_{i+1,j}\\ \;+(j+1)k^{-}\rho_{i,j+1}\;-\;(k_{i}^{+}+\;ik^{-}\;+\;k_{j}^{+}+\;jk^{-}\\ \;+k_{\text{L},\text{A}})\rho_{i,j},\;i,j=0,\ldots ,C \end{array}$$

(4) $$\begin{array}{r} k_{\text{L},\text{A}}=\;\{\;\begin{array}{c} k_{\text{A}},\text{if }i\;=\;j\;=\;C\\ k_{\text{L}}\text{ otherwise} \end{array},\\ \frac{d\rho_{\text{L}}}{dt}=\;k_{\text{L}}\sum_{i,j=0}^{C}\rho_{i,j}(1\;-\;\delta_{i,C}\delta_{j,C}), \end{array}$$

(5) $$\frac{d\rho_{\text{A}}}{dt}=\;k_{\text{A}}\rho_{C,C}\cdot$$

Here δ denotes the Kronecker delta function, which has value 1 when two indices have the same value and 0 otherwise. Note that equation (3) describes a situation where only one KC can attach to or detach from the spindle at a time, which can be used if KCs attach and detach independently of each other. We also introduce the average time of both prometaphase and metaphase, which we term the time of spindle assembly, $\langle t\rangle =\underset{0}{\overset{\infty }{\int }}t\frac{d\rho_{A}}{dt}dt/\underset{0}{\overset{\infty }{\int }}\frac{d\rho_{A}}{dt}dt$. Please note that the model does not take cell division into account and therefore the total number of cells is conserved.

## Results

### Chromosome loss in cells with one chromosome

To illustrate how chromosome loss occurs during the transition from prometaphase to anaphase, we numerically solve our model first for cells with only one chromosome, *C* = 1, for parameters given in Figure 1C. We discuss the time course for different populations of cells. Initially, cells have no chromosome attached to the spindle. In prometaphase, when spindle assembly starts and KCs attach to the spindle, the fraction of cells in this population decreases, while the fraction of cells in the other populations increases (compare the light and dark purple lines in Figure 1D). After an initial increase, the fraction of cells in prometaphase starts decreasing as more KCs attach, and cells switch to metaphase (compare purple and black lines in Figure 1D). Finally, cells switch to anaphase. The fractions of cells in anaphase increase and asymptotically approach a limit value because the model does not describe cells leaving anaphase (orange and blue lines in Figure 1D). In this case with only one chromosome, the fraction of cells with a lost chromosome is very low.

### Dramatic increase in the rate of chromosome loss with an increase in ploidy

**Figure 2:**
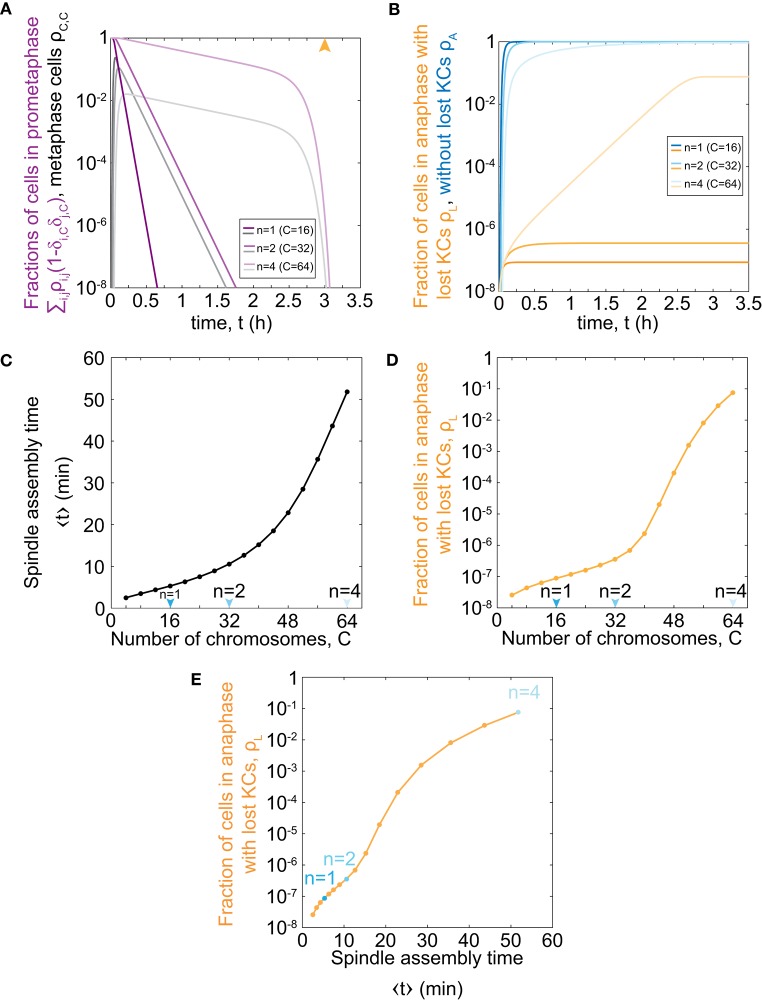
Model predictions for chromosome loss in cells of different ploidy. **(A)** Fraction of cells in prometaphase (purple) and metaphase (gray) for different numbers of chromosomes. The orange arrowhead denotes the value of the duration of mitotic arrest, *t*_0_. **(B)** Fraction of cells in anaphase with at least one KC unattached (orange) and in anaphase (blue). Three different shades in **(A,B)** correspond to different number of chromosomes, *C* = 16, 32, 64. For color-codes see inset legends. **(C)** Time of spindle assembly as a function of the number of chromosomes. **(D)** Rate of chromosome loss for cells as a function of the number of chromosomes. Arrowheads denote haploid, diploid and tetraploid number of chromosomes. **(E)** Rate of chromosome loss for cells as a function of the time of spindle assembly. Data points are obtained from **(C,D)**, and correspond to *C* = 4, …, 64. Cases with *C* = 16, 32, 64 are shown in blue. At *t* = 0, ρ_0,0_ = 1 and all other populations are 0. The other parameters are given in Figure 1C.

To explore the relevance of our model for haploid, diploid, and tetraploid yeast cells, we further solve our model for the respective number of chromosomes in each ploidy type, *C* = 16, 32, and 64 (Figure 2A). We find that cells with an increasing number of chromosomes spend a longer time in prometaphase and metaphase, though the general trend is similar to the case with *C* = 1 (Figure 1D). Additionally, there is a rapid decrease in the fraction of cells in prometaphase and metaphase, which occurs around the maximum time of mitotic arrest, *t* = *t*_0_, which is visible for cells with 64 chromosomes. After cells pass the maximum time of mitotic arrest, they predominantly enter anaphase regardless whether all KCs are attached. Thus, the more cells are still in prometaphase, the more cells will enter anaphase with unattached KCs. Because populations of cells with more chromosomes spend more time in prometaphase, they also enter anaphase later (Figures 2A,B). This time delay results in an increasing fraction of cells in anaphase with at least one lost KC because these cells have a greater chance to proceed to anaphase without a completely formed spindle (Figure 2B).

To explore which processes included in our model are responsible for significant chromosome loss, we determine the relevance of our model parameters. As our model describes both KC capture and transition to anaphase, we separately analyse the contribution of each process. We introduce the average time of both prometaphase and metaphase, which we refer to as the time of spindle assembly (Methods). We find that the time of spindle assembly increases with the number of chromosomes. Changing the chromosome number from 16 to 32 increases the time of spindle assembly approximately 2-fold, whereas, for a change from 32 to 64, it increases 5-fold (Figure 2C). Next, we explored how ploidy variations affect chromosome loss. We find that haploid (*C* = 16) and diploid (*C* = 32) cells have the same order of magnitude for the fraction of the population with at least one lost chromosome (Figure 2D). Interestingly, the fraction of cells with at least one lost chromosome increases dramatically for cells with higher ploidy, such as tetraploid cells (*C* = 64). When we plot the fraction of cells with lost kinetochores against spindle assembly time, we find that linear-scale changes in spindle assembly time result in exponential-scale changes in the rate of chromosome loss (Figure 2E). To summarize, our combined results show that small changes in spindle assembly time result in dramatic differences in the rate of chromosome loss as soon as prometaphase time approaches the maximum time of mitotic arrest.

### Relevance of parameters on the time of spindle assembly and the chromosome loss rate

**Figure 3:**
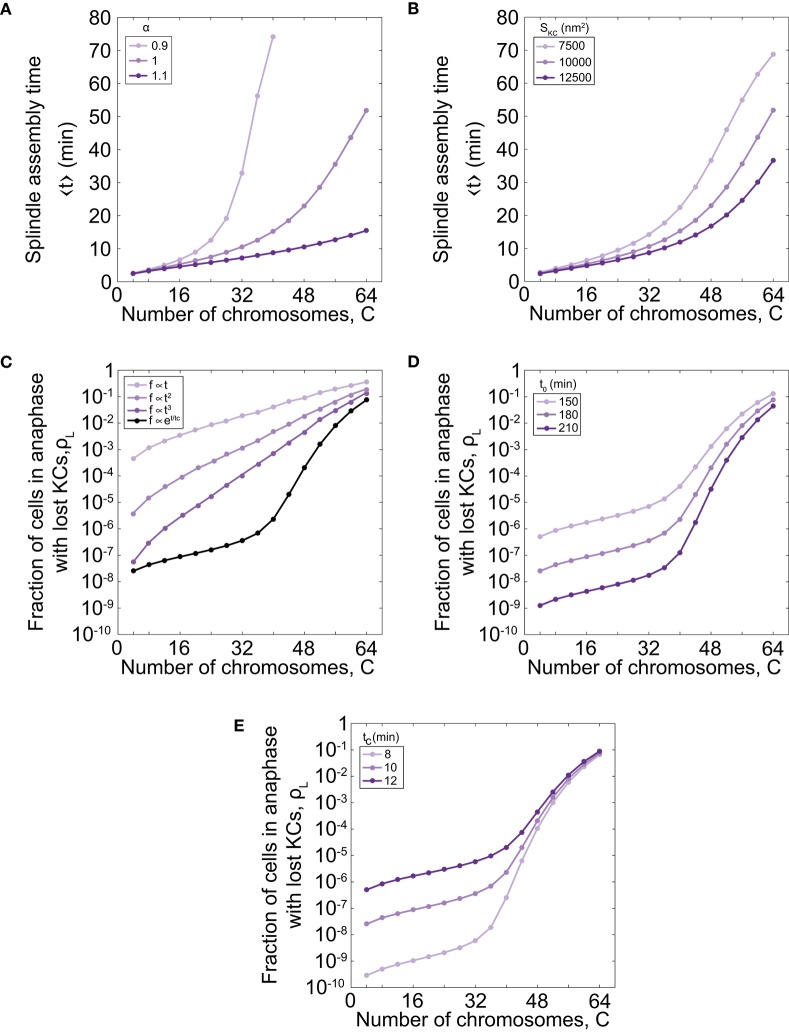
Time of spindle assembly and rate of chromosome loss for different number of chromosomes and different values of model parameters. **(A)** Time of spindle assembly for different number of chromosomes and three different values of α = 0.9, 1.0, 1.1. For color-codes see inset legend. The other parameters are given in Figure 1C. **(B)** The role of the cross-section area of the KC on the spindle assembly time. Three different shades correspond to different cross-section area of the KC, *S*_*KC*_ = 7500 nm^2^, 10000 nm^2^, 12500 nm^2^. For color-codes see inset legend. The other parameters are given in Figure 1C. **(C)** Rate of chromosome loss for different functional forms of the function *f*(*t*): linear function $f=\left(t_{c}/t_{0}^{2}\right)t$, quadratic function $f=\left(t_{c}/t_{0}^{3}\right)t^{2}$, cubic function $f=\left(t_{c}/t_{0}^{4}\right)t^{3}$, and exponential function *f* = *exp*[(*t* − *t*_0_)/*t*_*c*_]. For color-codes see inset legend. The other parameters are given in Figure 1C. **(D)** Rate of chromosome loss for different values of the parameter that describe the duration of mitotic arrest, *t*_0_. Three different shades correspond to different values of the parameter *t*_0_ = 150 min, 180 min, 210 min. For color-codes see inset legend. The other parameters are given in Figure 1C. **(E)** Rate of chromosome loss for different values of the characteristic timescale of mitotic arrest, *t*_*c*_. Three different shades correspond to different values of the parameter *t*_*c*_ = 8 min, 10 min, 12 min. For color-codes see inset legend. The other parameters are given in Figure 1C.

As our model describes spindle formation, we explore the relevance of parameters on the time of spindle assembly. We varied the parameter that links the number of chromosomes and microtubule nucleation sites, α , for different number of chromosomes. For parameter values α = 1.0 the time of spindle assembly increases with the number of chromosomes (Figures 2C, 3A). By increasing α to values >1 the assembly speeds up, but the influence is noticeable for a larger number of chromosomes (Figure 3A). By decreasing the parameter to the value α = 0.9 the assembly time dramatically increases with number of chromosomes and goes to infinity when there are more than 40 chromosomes. The infinite time of spindle assembly occurs for cells in which the number of microtubule nucleation sites at one pole is smaller than number of chromosomes. Interestingly, in yeast the value of the parameter α in cells is close to 1 (Figure 1C).

We next explore the relevance of geometry by varying the cross-section area of the KC, *S*_*KC*_. We find that geometry has a small contribution for a small number of chromosomes, but for larger number of chromosomes, the time of spindle assembly decreases with the increase of the cross-section area (Figure 3B). The role of the cross-section area occupied by one microtubule, *S*, can be inferred from these data because both parameters, the cross-section area occupied by one microtubule and the cross-section area of the KC, contribute to attachment probability *p*.

Further, we explore how the choice of the function that describes bypassing the checkpoint in mitotic arrest *f*(*t*) affects the chromosome loss rate. We find that for a linear function the chromosome loss rate increases as the number of chromosome increases (Figure 3C). However, in this case the model cannot explain experimental results quantitatively. For example, when number of chromosomes changes from 32 to 64 the chromosome loss rate increases approximately 20 times with the linear function, whereas when ploidy in experiments changes from diploid to tetraploid the loss rate increases thousand times. A chromosome loss rate in the model is more similar to the experimental results for nonlinear functional forms, such as quadratic and cubic functions (Figure 3C). Because from this analysis we cannot predict a functional form for the function *f*(*t*), we choose an exponential function as a simple function that provides agreement with experiments.

Finally, we explore how the parameters that describe bypassing the checkpoint in mitotic arrest, *t*_0_ and *t*_*c*_, affect the chromosome loss rate. We find that cells with shorter duration of mitotic arrest have an increased chromosome loss rate, irrespective of ploidy (Figure 3D). We also find that cells with a smaller characteristic timescale of mitotic arrest have a smaller rate of chromosome loss (Figure 3E).

## Discussion

Here we introduced a model in which we explored chromosome loss dynamics by accounting for key aspects of spindle assembly, including microtubule nucleation and KC attachment/detachment, together with a maximum time of mitotic arrest. Our theory provides a plausible explanation for experiments in yeast tetraploid cells, where there is a 1,000-fold increase in the rate of chromosome loss relative to haploid and diploid cells (Mayer and Aguilera, 1990; Storchová et al., 2006). Our model not only quantitatively predicts an increase in chromosome loss in cells with an increasing chromosome number, but also a longer duration of spindle assembly time. Indeed, the doubling time of yeast increases with ploidy in *S. cerevisiae*. For example, doubling times of haploid, diploid and tetraploid yeast cells in YPD is approximately 130, 146, and 171 min, respectively (Mable, 2001). This suggests that cells with increasing ploidy have an increased spindle assembly time, with differences in the same order of magnitude as in our model. However, this prediction needs to be further verified by direct measurements of average spindle assembly time in haploid, diploid, and tetraploid yeast cells. Key parameters of cytoplasmic microtubule dynamics were measured previously for diploid and tetraploid *S. cerevisiae* cells, including the rates of microtubule growth, shrinkage, catastrophe and rescue during G1 and mitosis (Storchová et al., 2006). We hypothesize that changes in these parameters may cause a change in the average spindle assembly time in a population of cells, but experimental validation in yeast is also needed.

In yeast cells of different ploidy, chromosome loss can occur for many reasons. Configurations with syntelic attachments can also appear and lead to chromosome loss. Storchova et al. detected an increased frequency of erroneous KC attachments in polyploid cells and suggest an important role for syntelic attachments based on increased activity of Ipl1, the yeast homolog of Aurora B (Storchová et al., 2006). Additionally, microtubules can detach from KCs during anaphase, which can further increase chromosome loss events. Thus, identifying experimentally which of these configurations are predominant in cells with lost chromosomes is crucial for establishing a complete picture of chromosome loss.

Laboratory tetraploid yeast cells have an increased rate of chromosome loss. However, a recent experimental evolution study with laboratory yeast cells found that some tetraploid cell lines could maintain their full chromosome complement (*C* = 64) for >1,000 generations (Lu et al., 2016). The evolved, stable tetraploid cells had elevated levels of the Sch9 protein, one of the major regulators downstream of TORC1, which is a central regulator of cell growth. Interestingly, the evolved stable tetraploid cells also had increased resistance to the microtubule depolymerizing drug benomyl relative to the ancestor tetraploid cells, indicating that increased Sch9 activity may, at least in part, rescue spindle formation defects observed in the ancestral tetraploid cells (Storchová et al., 2006; Lu et al., 2016). This is consistent with our model, where chromosome stability in tetraploid cells can be obtained by increasing the rate of spindle assembly.

This is the first theoretical study of the mechanism driving high rates of chromosome loss in polyploid yeast cells. Our approach for within-species ploidy variation can be applied to other species, including plants (Hufton and Panopoulou, 2009), where rates of chromosome loss are also higher in polyploid cells than in diploid cells, if the details of spindle self-organization are adjusted for the specific organism and cell-type. For example, for cells with more than one microtubule per KC, merotelic attachments need to be taken into account as well (Gregan et al., 2011). Future models will show the extent to which spindle assembly time influences the rate of chromosome loss for a variety of systems.

## Author contributions

NP, LL, and AS conceived the project. NP and LL developed the model, IJ solved the model. All authors wrote the paper.

### Conflict of interest statement

The authors declare that the research was conducted in the absence of any commercial or financial relationships that could be construed as a potential conflict of interest.
